# Disentangling ADHD's Presentation-Related Decision-Making—A Meta-Analytic Approach on Predominant Presentations

**DOI:** 10.3389/fpsyt.2021.519840

**Published:** 2021-02-18

**Authors:** Marcel Schulze, David Coghill, Silke Lux, Alexandra Philipsen

**Affiliations:** ^1^Department of Psychiatry and Psychotherapy, University of Bonn, Bonn, Germany; ^2^Department of Pediatrics, University of Melbourne, Melbourne, VIC, Australia; ^3^Department of Psychiatry, University of Melbourne, Melbourne, VIC, Australia; ^4^Murdoch Children's Research Institute, Melbourne, VIC, Australia

**Keywords:** attention deficit and hyperactivity disorder, decision making, meta-analysis, inattention and hyperactivity, risk behavior

## Abstract

**Background:** Deficient decision-making (DM) in attention deficit/hyperactivity disorder (ADHD) is marked by altered reward sensitivity, higher risk taking, and aberrant reinforcement learning. Previous meta-analysis aggregate findings for the ADHD combined presentation (ADHD-C) mostly, while the ADHD predominantly inattentive presentation (ADHD-I) and the predominantly hyperactive/impulsive presentation (ADHD-H) were not disentangled. The objectives of the current meta-analysis were to aggregate findings from DM for each presentation separately.

**Methods:** A comprehensive literature search of the PubMed (Medline) and Web of Science Database took place using the keywords “ADHD,” “attention-deficit/hyperactivity disorder,” “decision-making,” “risk-taking,” “reinforcement learning,” and “risky.” Random-effects models based on correlational effect-sizes were conducted. Heterogeneity analysis and sensitivity/outlier analysis were performed, and publication biases were assessed with funnel-plots and the egger intercept.

**Results:** Of 1,240 candidate articles, seven fulfilled criteria for analysis of ADHD-C (*N* = 193), seven for ADHD-I (*N* = 256), and eight for ADHD-H (*N* = 231). Moderate effect-size were found for ADHD-C (*r* = 0.34; *p* = 0.0001; 95% CI = [0.19, 0.49]). Small effect-sizes were found for ADHD-I (*r* = 0.09; *p* = 0.0001; 95% CI = [0.008, 0.25]) and for ADHD-H (*r* = 0.1; *p* = 0.0001; 95% CI = [−0.012, 0.32]). Heterogeneity was moderate for ADHD-H. Sensitivity analyses show robustness of the analysis, and no outliers were detected. No publication bias was evident.

**Conclusion:** This is the first study that uses a meta-analytic approach to investigate the relationship between the different presentations of ADHD separately. These findings provide first evidence of lesser pronounced impairment in DM for ADHD-I and ADHD-I compared to ADHD-C. While the exact factors remain elusive, the current study can be considered as a starting point to reveal the relationship of ADHD presentations and DM more detailed.

## Introduction

ADHD is a neurodevelopmental disorder associated with inappropriate levels of inattention and/or hyperactivity/impulsivity (1). Subtypes are categorized depending on the degree of inattention and/or hyperactivity in predominantly inattentive presentation (ADHD-I), predominantly hyperactive/impulsive presentation (ADHD-H), and the combined presentation (ADHD-C) (2). ADHD as a disorder has been considered by some to be primarily a disorder of executive dysfunction (3). Among these, dysfunction in inhibition, working memory, and task switching are most consistently reported (4). However, the relationship between executive dysfunction and ADHD is not that simple because not all of those with ADHD exhibit executive dysfunction (5). There are now several other conceptualizations that address this heterogeneity and propose multiple pathway models (5, 6). According to these models, ADHD can arise from dysfunction across several different pathways that include executive and non-executive dysfunctions as well pathways that emphasize motivational aspects accompanied with suboptimal reward processes, delay aversion, that is, the drive toward immediate reinforcement, and to escape the negative affect induced by delay (7). These latter two pathways are mediated by deficits on mesocortical control circuits for the cognitive pathway and by alterations in meso-limbic reward circuits for the motivational pathway.

Decision-making (DM) can be viewed as choosing one specific action among others after evaluating the potential outcomes, preferences, and context. This encompasses scenarios ranging from simple perceptual decisions to complex learned situations (e.g., reinforcement learning) as well as risky DM, all of which have been studied empirically (8, 9). To optimally decide for the best outcome, an interplay of cognitive functions have to take place. These comprise of self-referential processes, for example, reflections on autobiographical past and prospection about possible future events. Further, working memory, inhibition, and planning, as well as value estimation, outcome appraisal, and learning need to work together, further underline the complexity of the DM process. However, a decision does not necessarily always depend on all aforementioned cognitive functions. Dependent on the task, different types of DM can be distinguished: those who predominantly require “cool aspects” of cognitive control mediated by dorsolateral prefrontal cortex (DLPFC) can be differentiated by those scenarios predominantly requiring affect regulation, that is, motivational aspects mediated by ventral medial prefrontal cortex (VMPFC) (4, 10, 11). Tasks involving cool aspects of DM are the Cambridge gambling task, game of dice task, make-a-match game, and probabilistic discounting task. All these tasks share the feature that a fast-intuitive strategy can be applied, since the learned outcome is based on associations (4, 12, 13). Tasks involving hot aspects are, for example, the Iowa gambling task, balloon analog risk task, door-opening task, and card-playing task. These tasks are solved best by applying a slow analytical strategy, that is, based on rule learning. Cool aspects of DM are deficient in ADHD in terms of less rational choices and lower risk adjustment compared to controls (14). Also, when the task is more progressed and the participants become more used to it, those with ADHD perform worse than controls, that is, riskier (15). In tasks that involve more analytical rule learning, for example, the Iowa gambling task, children/adolescents with ADHD show more risky behavior, choose less often the advantageous decks compared to controls, and are sensitive to the frequency and not to the magnitude of a punishment (16). However, there are studies showing no differences in terms of risky behavior and the amount of choices for the advantageous decisions. Further, ADHD-subtype comparisons revealed no differences. Other tasks, for example, the door-opening task and the balloon analog risk task, show higher risk-taking behavior in ADHD. While Groen et al. reported weaker evidence for adults compared to children/adolescents, no age moderation effect was reported in a meta-analytic study by Groen et al. (14) and Jackson and MacKillop (17). In their case-control delay discounting the meta-analytic model, an effect-size (ES) of d = 0.43 was reported (17). Another meta-analysis reported an odd ratio of 1.9 for single choice paradigms (i.e., choose one option among two rewards with different size and delay to delivery) and standardized mean difference for temporal discounting paradigms of 0.43 for ADHD vs. controls (18). In conclusion, patients with ADHD show deficient DM on both the cool and hot aspects of executive functioning. Despite methodological differences in task design and heterogeneity in study samples, the effects found appear to be relatively robust throughout development in ADHD. We are currently lacking a systematic investigation of the single presentations since reviews and meta-analyses and most of the single studies have considered all groups in one analysis and do not differentiate between the different ADHD presentations (14, 17). Some studies have reported that ADHD subtypes differ in their correlation to DM (19, 20). Since there is no analysis available, the aim of the current review is to provide a quantitative overview of DM in the ADHD-I, ADHD-H and, ADHD-C separately. We hypothesize stronger ESs for the ADHD-C presentation.

## Methods

### Search Strategies

**Figure 1:**
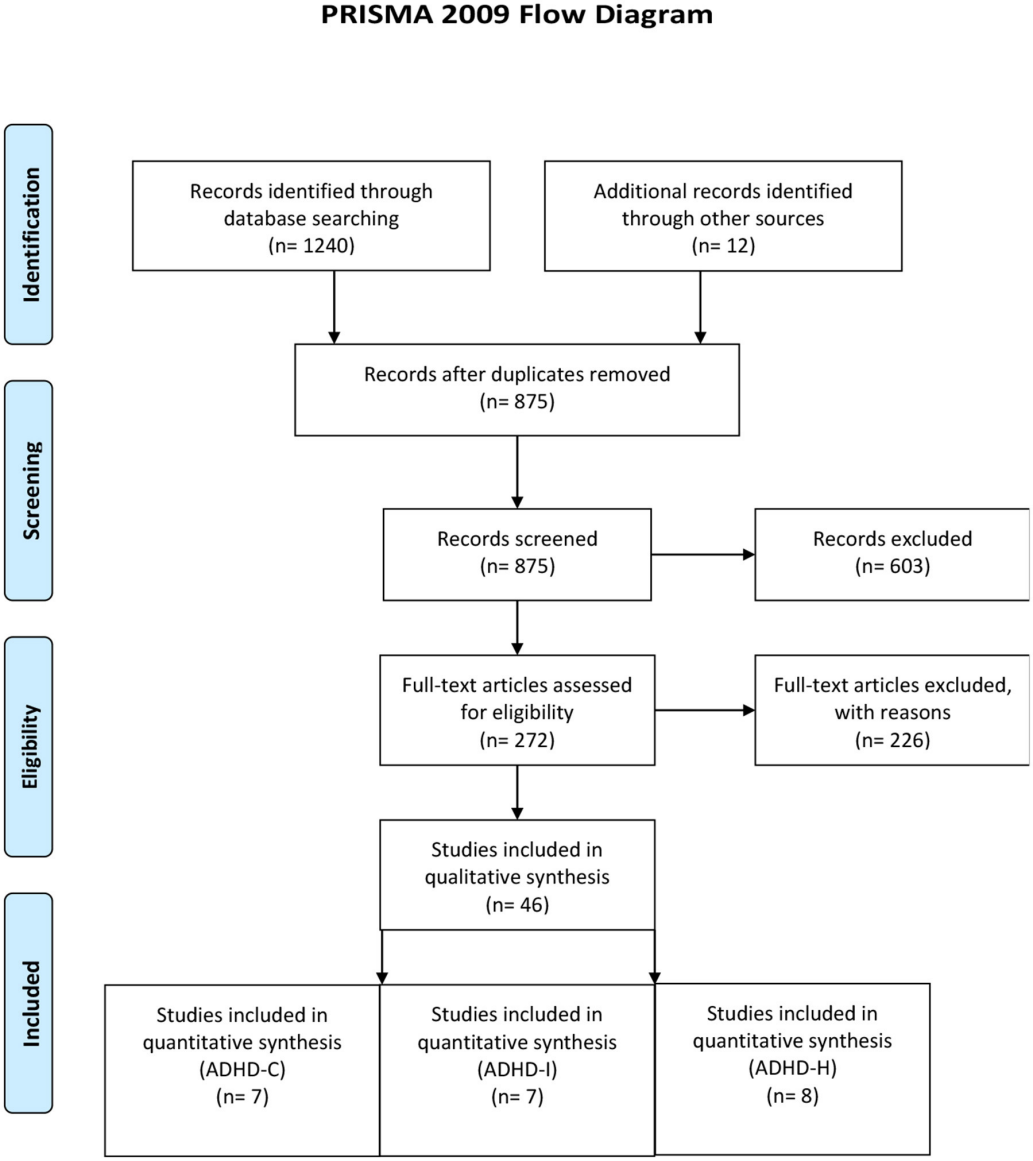
Prisma flow diagram.

This study followed the guidelines of the Preferred Reporting Items for Systematic Reviews and Meta-Analyses (PRISMA) (Figure 1) to achieve a high standard of reporting (21). A comprehensive literature search of the PubMed (Medline) and Web of Science Database took place using the keywords “ADHD,” “attention-deficit/hyperactivity disorder,” “decision-making,” “risk-taking,” “reinforcement learning,” and “risky” in all possible combinations. The search was conducted in July 2020 with no time interval specified. Reference lists of obtained articles were also considered.

### Study Selection

**Figure 2:**
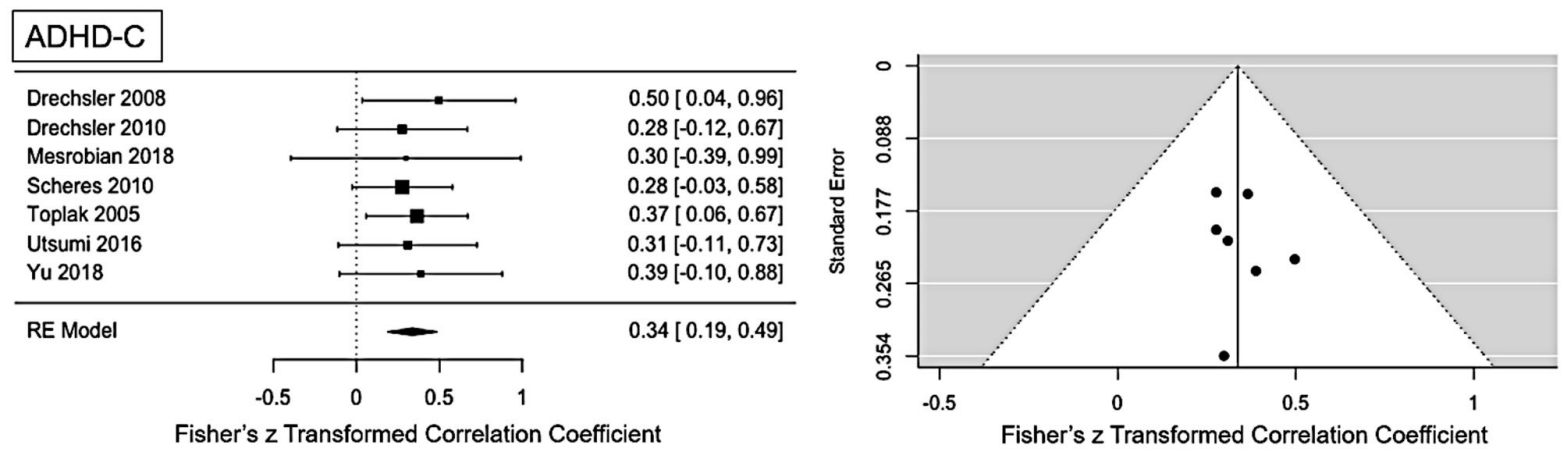
Forest plot of the effect-sizes and 95% confidence intervals for ADHD-combined presentation.

Studies were included if they met the following criteria: (1) a publication of the research paper in a peer-reviewed journal, (2) standardized ADHD-diagnostic/assessment procedures (i.e., structured or semi-structured interviews or ADHD-specific questionnaires) according to DSM IV/V [see (22, 23) for an overview], (3) tasks including risk-taking behavior, rewards and DM, and (4) comparison to a healthy, typically developing group. Studies were excluded if (1) the predominant presentations of ADHD subtypes were not considered in the analysis or (2) ESs were not reported. See Figure 2 for a schematic overview of study inclusion/exclusion.

### Recorded Variables

The recorded variables from each study were: sample size, mean age of the participants, type of control group, kind of DM-task, and metric for calculation of ES. Data were extracted from each study by one of the authors (MS) and checked by another author (SL) to minimize data selection errors.

### Meta-Analytic Approach

To calculate a random-effects model, correlational ESs were extracted. If only *F*-or *t*-values were provided, correlation coefficients were obtained according to the following formula (24):

$$\begin{array}{l} r=\frac{t}{\sqrt{t^{2}+N-2}} \end{array}$$

If partial eta squared were provided, it was transformed to Cohens' f/Cohens' d and finally to the correlation coefficients using the following formula (25, 26):

$$\begin{array}{lll} f\; & = & \;sqr\left(\;eta^{\wedge }2/\left(\;1\;-\;eta^{\wedge }2\right)\right)\\ r & = & \frac{d}{\sqrt{d^{2}}+a} \end{array}$$

where, a is a correction factor for cases of unbalanced numbers of participants between the groups (27). The meta-analytic procedure was realized using R-software library package metafor [version 2.0-0 (28)]. Sampling variances weighted by sample size were determined by Hunter and Schmidt method because this method estimates the average correlation with the least error with comparative accuracy as, for example, Hedges and Vevea (29). Heterogeneities were assessed with Q and I2 statistics (30). Conventions were followed by the interpretation of I2: values of 0.25, 0.50, and 0.75 correspond to low, moderate, and high between-trial heterogeneities (30). To evaluate the influence of an individual study on the overall effect, leave-one-out analysis (repeat the ES-calculation while omitting a study at a time) using dmetar was performed (31). Outlier detection was performed using “find.outlier” -function in dmetar. In the case of the presence of outlier, the study was excluded, and the ES-calculation was repeated. The publication bias was assessed with funnel-plots and an egger intercept. Since study inclusion is not determined by age, age was included as a moderator variable in a mixed-effects model if heterogeneity is present.

## Results

### Included Studies and Sample Characteristics

The literature search resulted in a final set of seven studies for ADHD-C (mean age: 12.9, SD: 4.7), eight studies for ADHD-H presentation (man age: 12.2, SD: 4.7), and seven studies for ADHD-I presentation (mean age: 12.5, SD: 5.0) (see search flow diagram in Figure 2). Total sample size comprised of 193 patients for ADHD-C presentation, 265 patients for ADHD-I, and 231 patients for ADHD-H presentation. Except for one study, where performance of DM was compared to oppositional defiant disorder (32), all studies used a healthy age-matched control group. The following paradigms were applied: temporal discounting task (19, 33, 34), Iowa gambling task (32), game of dice task (35), make a match game (15), probabilistic game task (36), and card task (37) (see Table 1 for an overview).

**Table 1:** Study-Overview.

**Study**	**Patients (f)**	**Mean-age (Std)**	**DM task**	**Control-group**	**Diagnostical instrument**
Bubier and Drabick (32)	63 (27)	7.79 (1.08)	Iowa Gambling Task	ODD	Child Symptom Inventory-4
Drechsler et al. (35)	21 (2)	12.2 (0.8)	Game of Dice Task	Healthy	Conners' Teacher Rating Scale, SNAP
Drechsler et al. (15)	28 (8)	9.2 (1.2)	Make a Match Game	Healthy	Swanson, Nolan, and Pelham rating scale
Mesrobian et al. (36)	18 (11)	22.3 (0.7)	Probabilistic Game Task	Healthy	Conners' Adult ADHD Rating Scales-Self Report, adult ADHD Self-report Scale
Scheres et al. (19)	45 (14)	11.4 (3.4)	Temporal Discounting Task	Healthy	Conners Parent Rating Scale, Schedule for Affective Disorders and Schizophrenia for School-Age Children
Toplak et al. (37)	44 (6)	15.6 (1.4)	Cardtask	Healthy	Schedule for Affective Disorders and Schizophrenia for School-Age Children
Utsumi et al. (34)	25 (6)	10.28 (1.30)	Temporal Discounting Task	Healthy	Child Behavior Checklist, Brazilian version of the Conners Rating Scale
Yu et al. (33)	19 (1)	9.1 (0.7)	Temporal Discounting Task	Healthy	Conners' Teacher Rating Scale, Conners' Parent Rating Scale–Revised

*f, female; Std, standard deviation; DM, decision making; ODD, oppositional defiant disorder*.

### Meta-Analytic Findings

#### ADHD-C Presentation

Moderate ES for ADHD-C presentation was found: *r* = 0.34 (95% CI = [0.19, 0.49], *p* = <0.001). No significant heterogeneity is present (Q = 0.81, *p* = 0.99, *I*^2^ = 0%). Visual inspection of the funnel-plots and the egger intercept (z = 0.2, *p* = 0.84) suggesting the absence of a publication bias (see Figure 2). Influence analysis, that is, leave one out sensitivity analysis, showed that the result is not driven by a single study (see **Figure 5**). No outlier was identified.

#### ADHD-H Presentation

**Figure 3:**
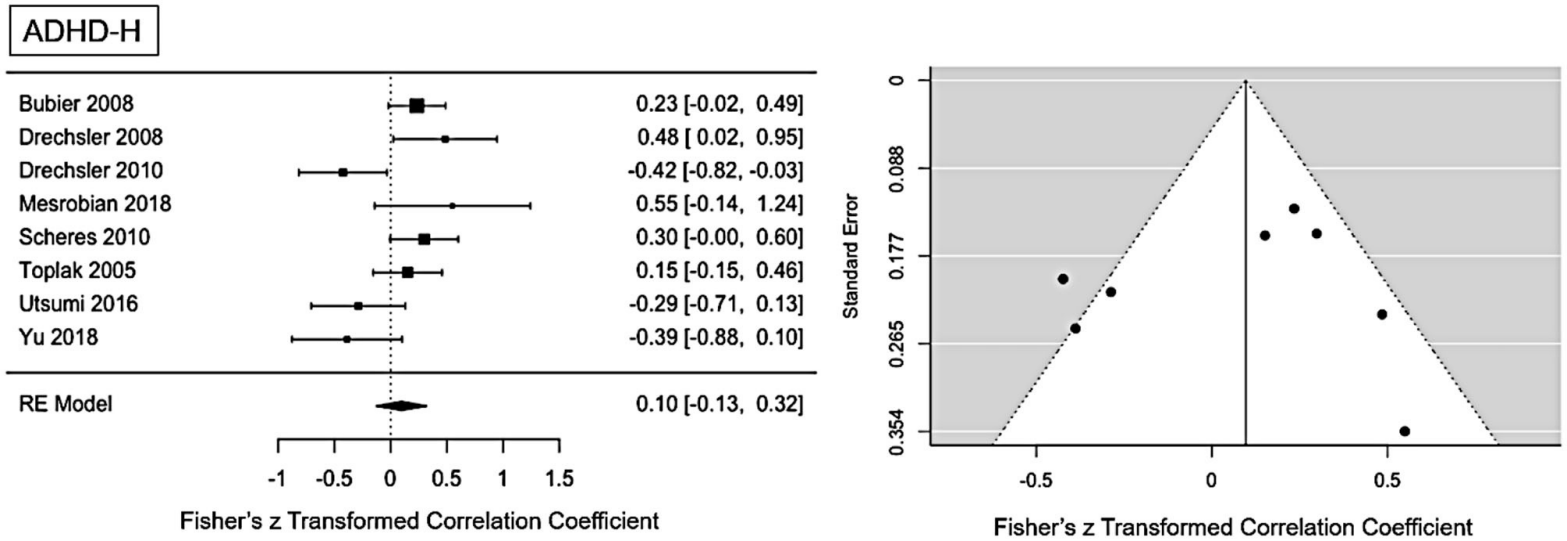
Forest plot of the effect-sizes and 95% confidence intervals for ADHD-hyperactivity/impulsive-presentation.

The main ES for the ADHD-H presentation was *r* = 0.1 (95% CI = [−0.012, 0.32], *p* = 0.0001). The forest plot with the within-study ES and aggregated ES is presented in Figure 3. Moderate heterogeneity is present in the study sample (Q = 21.1, *p* = 0.003, *I*^2^ = 61.02%, df = 7). Since we included pediatric and adult studies in the analysis, we performed a moderator analysis with age, suggesting that age has no influence on the heterogeneity, Q(df = 1)=1.24, *p* = 0.26. Visual inspection of the funnel-plots and the egger intercept (z = −0.47, *p* = 0.64) suggesting the absence of a publication bias (see **Figure 5** for the funnel-plots). Influence analysis, that is, leave-one out analysis, suggests general robustness of the result. Outlier analysis could not detect any outlier in the current analysis.

#### ADHD-I Presentation

**Figure 4:**
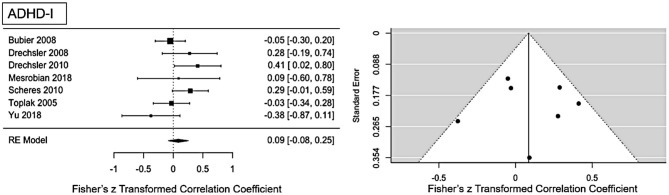
Forest plot of the effect-sizes and 95% confidence intervals for ADHD-inattention-presentation.

**Figure 5:**
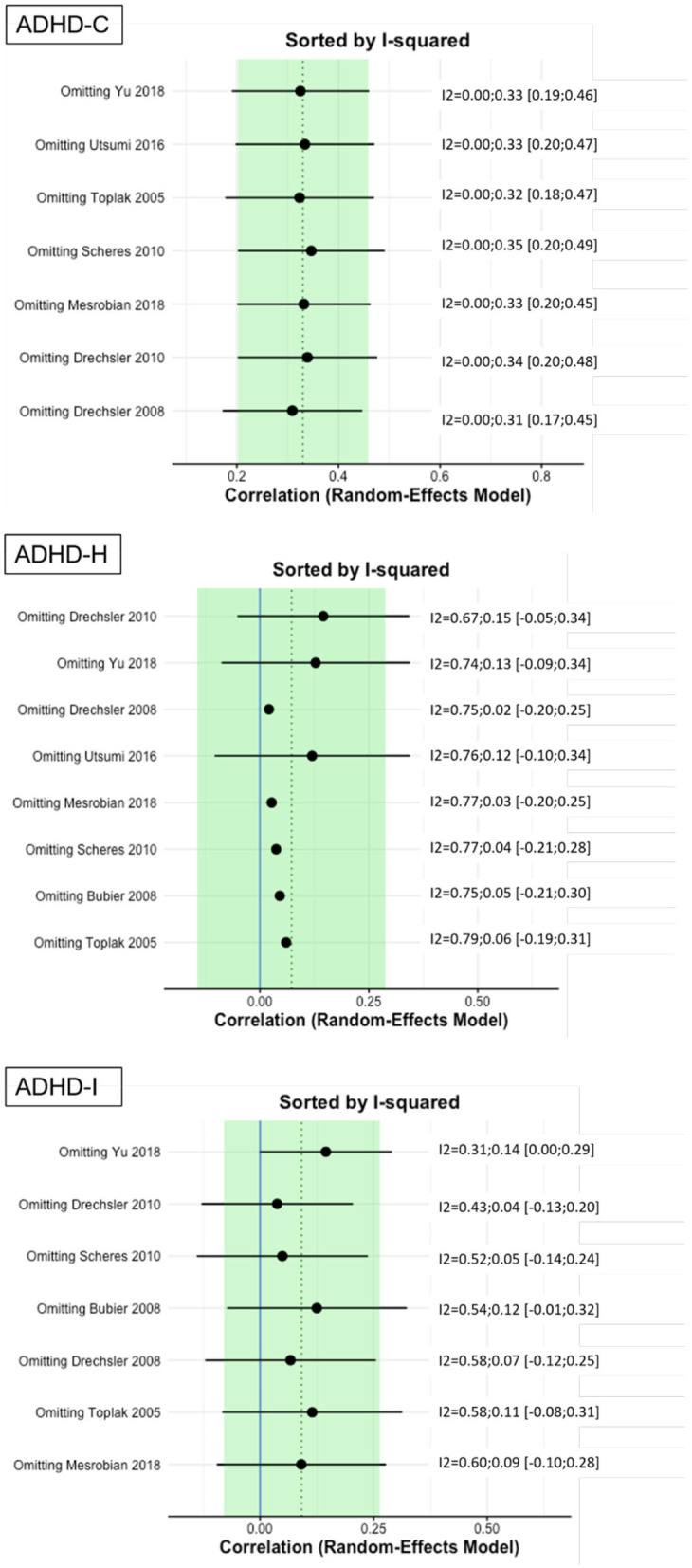
Results of leave-one-out analysis.

For ADHD-I presentation an ES of *r* = 0.09 (95% CI = [0.008, 0.25], *p* = 0.0001) was found (see Figure 4 for forest plot). Low heterogeneity is present in this sample (Q = 10.1, *p* = 0.12, *I*^2^ = 29.59%, df = 6). No publication bias is evident as visual inspection of the funnel-plots (see Figure 5) and Eggers intercept (z = 0.11, *p* = 0.9) suggest. Moderator with age as regressor show no moderate influence, Q(df = 1) = 0.03, *p* = 0.85. Influence analysis showed general robustness of the results, and no outlier was detected.

## Discussion

Since the majority of published studies report associations of DM and ADHD-C presentation, the aim of the current study was to investigate DM separately for the ADHD-C, ADHD-I, and ADHD-H presentation. Moderate ES was found for ADHD-C (*r* = 0.34), small ES for ADHD-H (*r* = 0.1), and ADHD-I (*r* = 0.09). Heterogeneity was moderate in ADHD-H and low for ADHD-I and ADHD-C. In all analyses, no outlier studies were evident. The ES for ADHD-C were comparable to other meta-analyses, which also report medium ES, for example, d = 0.43 (17), SMD = 0.43, and O*R* = 1.9 (18). For the first time, we report ES for the predominant presentations separately. The smaller ES for ADHD-I and ADHD-H might be a hint that these presentations are less impaired dependent on the context DM is needed. According to Sonuga-Barke's dual pathway model, ADHD is associated with deficits on the motivational and cognitive/executive pathways. More specifically, the model proposes that delay aversion and poor inhibitory control are independent coexisting characteristics of ADHD. Deficient delay aversion is mediated by the mesolimbic structures that are associated with dopaminergic reward circuits (e.g., nucleus accumbens). Poor inhibitory control is mediated by aberrant connectivity from mesocortical structures to the prefrontal cortex (6). Delay aversion and poor inhibitory control are differentially expressed dependent on the DM type. When confronted with a delay, ADHD patients with delay aversion react with a negative emotional response and tend to choose the impulsive element of a decision, that is, the smaller sooner reward over a larger longer reward (38). In scenarios, where the delay cannot be overcome with an impulsive choice, the perception might switch away from the task and to the environment. In such delay situations, an increase in general activity and inattention arise (38, 39). As suggested by Sonuga-Barke (38), the context of delay-rich scenarios decides whether ADHD patients may be a delay-averse subtype (hence, reacting with impulsivity), whereas more pervasive expressions might reflect other executive deficits associated with inhibitory dysfunction.

In our sample, three studies used delay-rich paradigms, that is, temporal discounting (19, 33, 34), whereas the other studies modulated the height of an immediate reward dependent on the decision made. In delay-rich scenarios, the ADHD-C presentations seem to show more deficient DM-performance compared to ADHD-I and ADHD-C. In other words, ADHD-C presentations may be more sensitive to delay-rich scenarios; hence, it may be more associated with a delay-averse subtype as suggested by Sonuga-Barke. When the delay is rather small as in Scheres et al. (19), where a small delay of 60 s is used, the difference in DM-performance between the ADHD presentations decreases. Future studies are needed to explore the specific effect of the delay-length on ADHD-presentations. Delay-discounting paradigms are associated with hot executive functions that encompass emotions, motivation, and the interplay of desire for an immediate gratification and long-term rewards (40). Carefully interpreted, the delay in DM scenarios may have higher impact on performance in ADHD-C presentations compared to ADHD-I/H presentations. The cool aspects of executive functions are associated with slow, analytical strategies and are dependent of inhibition, planning, and working memory (40). In our analysis, different paradigms were used that rely on cool executive functions: game of dice task (35), make-a-match game (15), and probabilistic-game tasks (36). While the ADHD-I presentation was associated with the smallest ES for the game of dice task (*r* = 0.28) and probabilistic-game task (*r* = 0.09), it was also associated with highest ES for the make-a-match task (*r* = 0.41). These differences could be due to diagnostical differences, that is, used instruments and experience in rating ADHD-symptoms across studies (22). Alternatively, the paradigms used in the studies may differ in the requirements of executive functions, for example, the game of dice task and the probabilistic-game task rely more on guessing the outcome and may be lower working memory demanding. In contrast, the make-a-match game, where two matching cards have to be found, needs higher working memory capacity.

According to the neuroeconomic model proposed by Sonuga-Barke and Fairchild (41), deficient DM in ADHD can be mediated by disruptions in three neuronal subsystems, which interact with each other: disrupted connectivity within the default mode network, dorsal-frontostriatal activations, and dopamine dysregulation in ventral frontostriatal networks (7, 41). In brief, an aberrant connectivity pattern in the default-mode network is associated with impaired self-referential thoughts, poor goal setting/implementation, and unstable value hierarchies. Also, impaired cognitive functioning is associated with deficient DLPFC-activation (part of the dorsal frontostriatal system), as for instance difficulties in updating working memory about different choice options. Impairments in the ventral frontostriatal network are associated with prospective behavior, that is, predicting future rewards. Taken together, DM in ADHD can be associated with impairment in the different functions necessary for DM. Distinctive neuronal activation patterns between ADHD-C and ADHD-I presentations compared to healthy controls could be differentiated. While both ADHD-I and ADHD-H presentations have been characterized by atypical connectivity throughout the brain, the combined presentation has been associated with more deviant connectivity in the default-mode network (42). In terms of DM, this could mean that the degree of impairment of the default-mode network in the ADHD-I and ADHD-H presentation might not result in the same behavioral consequences compared to ADHD-C presentation. This may lead to better goal setting/implementation and more stable value hierarchies in ADHD-I/H, compared to ADHD-C presentation. Further, ADHD-I has been shown to be associated with aberrant activation in dorsolateral prefrontal regions, which is proposed as a region that is important for cognitive functioning, for example, inhibition, working memory, and planning in the neuroeconomic model. To address the relationship of ADHD presentations, DM, and core separable neuropsychological functions, such as working memory, inhibition, and sustained attention, it seems prudent to calculate ES between DM and neuropsychological functions and between ADHD symptoms and neuropsychological functions, respectively. However, most of the included studies did not provide appropriate measurements, and therefore it was not possible to extract metrics in a sufficient manner for a proper ES calculation.

The relationship between neuropsychological functioning and ADHD symptoms seem not to be as directly dependent on each other as usually often assumed. Based on heterogeneity of impairment in cognitive functions, studies have identified distinct ADHD groups (independent of ADHD presentations). While some patients do show intact neuropsychological functioning in some aspects of cognition, others do not appear to have any cognitive deficits in commonly assessed domains (5, 43). Coghill and colleagues have proposed a model that considers that ADHD symptoms do not arise as a direct consequence of cognitive deficits rather that symptoms and cognition are relatively independent constructs with their causal roots in distinct aspects of brain structure and function (44). Therefore, it will be important to investigate the different neuropsychological functions needed for DM separately in the three different ADHD presentations. It will also be important to investigate the ways that the different ADHD presentations and their associated patterns of DLPFC activation, as well as frontostriatal systems (as mentioned above) contribute to problems with DM.

## Limitations

Since the majority of published studies include mostly the combined type or fail to differentiate between the different ADHD presentations, the number of included studies in the current analysis and the total sample size is rather small. In future studies, researchers should include data relating to the different ADHD presentations and provide metrics on the association of ADHD presentations and DM. Another limitation is the absence to control for diagnostical instruments used to assess ADHD symptoms. There may be certain variability in the diagnostical validity of the symptoms due to the use of different diagnostical tools and also the experience of the staff to rate symptoms for interview-based assessments. Unfortunately, due to the limited number of included studies, we could not assess the impact of the used diagnostical instruments. Further, ADHD subtypes are not discrete entities that are constant over time. When followed over time, patients may switch from one subtype to another, for example, from ADHD-C to ADHD-I (45, 46). This suggests when looking at DM in ADHD, subtype classification might not necessarily be the primary factor for a differentiation of DM performance across patients. We argue to also consider, for example, neuropsychological scores and comorbidities for a more comprehensive analysis. Further, the number of subjects per ADHD presentation might be imbalanced in the original studies, considering the prevalence of each ADHD presentation (47). Another limitation is that most of the studies (32, 37) include DM tasks that require cool aspects of executive functioning. It cannot be ruled out whether the current results are biased for this type of DM tasks. The small sample size hindered subanalysis on task designs that could potentially separate tasks requiring cool and hot aspects.

## Conclusion

This is the first study to use a meta-analytic approach to investigate the relationship of ADHD-C, ADHD-I, and ADHD-H presentations in DM separately. While meta-analytic evidence in the literature shows deficient DM in ADHD-C presentations, it appears less clear in comparison to ADHD-I and ADHD-H presentations. The current meta-analysis provides rather limited evidence, but cautiously interpreted, it might be that patients with ADHD-I and ADHD-H presentations show less impairment in DM skills. However, the interplay between the triad of ADHD-presentation-specific symptoms, DM skills and neuropsychological functions are complex and not yet fully understood. The current study is considered as a starting point to clarify the relationship of ADHD presentations and DM. Since the current evidence is rather limited, future work is needed to support our findings and clarify the interplay.

## Data Availability Statement

The datasets analyzed in this article are not publicly available. Requests to access the datasets should be directed to marcel.schulze@ukbonn.de.

## Author Contributions

MS and SL: literature search, figures, data analysis, data interpretation, and writing. DC and AP: data interpretation, writing, and supervision. All authors contributed to the article and approved the submitted version.

## Conflict of Interest

The authors declare that the research was conducted in the absence of any commercial or financial relationships that could be construed as a potential conflict of interest.
